# Structural characteristics of alkaline phosphatase from the moderately halophilic bacterium *Halomonas* sp. 593

**DOI:** 10.1107/S1399004713033609

**Published:** 2014-02-22

**Authors:** Shigeki Arai, Yasushi Yonezawa, Matsujiro Ishibashi, Fumiko Matsumoto, Motoyasu Adachi, Taro Tamada, Hiroko Tokunaga, Michael Blaber, Masao Tokunaga, Ryota Kuroki

**Affiliations:** a Quantum Beam Science Directorate, Japan Atomic Energy Agency, 2-4 Shirakata-shirane, Tokai, Ibaraki 319-1195, Japan; b Applied and Molecular Microbiology, Faculty of Agriculture, Kagoshima University, 1-21-24 Korimoto, Kagoshima 890-0065, Japan; c College of Medicine, Florida State University, 1115 West Call Street, Tallahassee, FL 32306-4300, USA

**Keywords:** halophilic enzymes, alkaline phosphatase

## Abstract

Alkaline phosphatase (AP) from the moderate halophilic bacterium *Halomonas* sp. 593 (HaAP) catalyzes the hydrolysis of phosphomonoesters over a wide salt-concentration range (1–4 *M* NaCl). In order to clarify the structural basis of its halophilic characteristics and its wide-range adaptation to salt concentration, the tertiary structure of HaAP was determined by X-ray crystallography to 2.1 Å resolution. The unit cell of HaAP contained one dimer unit corresponding to the biological unit. The monomer structure of HaAP contains a domain comprised of an 11-stranded β-sheet core with 19 surrounding α-helices similar to those of APs from other species, and a unique ‘crown’ domain containing an extended ‘arm’ structure that participates in formation of a hydrophobic cluster at the entrance to the substrate-binding site. The HaAP structure also displays a unique distribution of negatively charged residues and hydrophobic residues in comparison to other known AP structures. AP from *Vibrio* sp. G15-21 (VAP; a slight halophile) has the highest similarity in sequence (70.0% identity) and structure (C^α^ r.m.s.d. of 0.82 Å for the monomer) to HaAP. The surface of the HaAP dimer is substantially more acidic than that of the VAP dimer (144 exposed Asp/Glu residues *versus* 114, respectively), and thus may enable the solubility of HaAP under high-salt conditions. Conversely, the monomer unit of HaAP formed a substantially larger hydrophobic interior comprising 329 C atoms from completely buried residues, whereas that of VAP comprised 264 C atoms, which may maintain the stability of HaAP under low-salt conditions. These characteristics of HaAP may be responsible for its unique functional adaptation permitting activity over a wide range of salt concentrations.

## Abstract 2

In order to clarify the structural basis of the halophilic characteristics of an alkaline phosphatase derived from the moderate halophile *Halomonas* sp. 593 (HaAP), the tertiary structure of HaAP was determined to 2.1 Å resolution by X-ray crystallography. The structural properties of surface negative charge and core hydrophobicity were shown to be intermediate between those characteristic of halophiles and non-halophiles, and may explain the unique functional adaptation to a wide range of salt concentrations.

## 1. Introduction   

Halophilic bacteria live and grow in high-salt environments, and are classified into essentially three groups (slight halophiles, moderate halophiles and extreme halophiles; Mishra & Champagne, 2009 ▶). These halophiles exhibit different optimal salt concentrations for growth as follows: 2–5% salt for slight halophiles (seawater contains ∼3.5% salt), 5–20% salt for moderate halophiles and >20% (up to saturated salt solutions of 36–39% depending on temperature) for extreme halophiles (Hayashi *et al.*, 1973 ▶; Vreeland & Hochstein, 1992 ▶; Ollivier *et al.*, 1994 ▶; Seckbach, 2001 ▶). To adapt to an environment containing almost saturated salt, proteins from extreme halophiles and from the extracellular and periplasmic fractions of moderate halophiles exhibit abundant acidic surface amino acids (*e.g.* Glu, Asp) that promote solubility in a high-salt environment (Lanyi, 1974 ▶). Halophile proteins typically also exhibit a reduced core hydrophobicity, as salt stabilizes such interactions (Paul *et al.*, 2008 ▶); thus, an abundance of negative surface charge and reduced core hydrophobicity are two of the characteristic features of halophile proteins in comparison to non-halophiles and other extremophiles.

Alkaline phosphatases (EC 3.1.3.1) are periplasmic enzymes that are found in organisms ranging from bacteria (including extremophiles) to mammals. As of 2013, 66 crystal structures of AP have been deposited in the Protein Data Bank (PDB). Among these structures, APs from the extreme halophile *Halobacterium salinarum* (HsAP; PDB entry 2x98; Wende *et al.*, 2010 ▶) and from the slight halophiles *Vibrio sp.* G15-21 (VAP; PDB entry 3e2d; Helland *et al.*, 2009 ▶) and the Antarctic bacterium TAB5 (TAP; PDB entry 2iuc; Rina *et al.*, 2000 ▶; Wang *et al.*, 2007 ▶; Koutsioulis *et al.*, 2010 ▶) have been reported. The ratios of acidic to basic residues [(Asp + Glu)/(Arg + Lys)] are 63/27 for HsAP (an extreme halophile), 67/51 for VAP (a slight halophile) and 42/35 for TAP (a slight halophile) (Ishibashi *et al.*, 2005 ▶). The amino-acid sequences of these APs show relatively low similarity (∼30%), but their tertiary structures are similar: the sequence identity and r.m.s.d. for C^α^ atoms of the monomer are 31% and 1.95 Å between HsAP and VAP, 33% and 1.34 Å between HsAP and TAP, and 35% and 1.32 Å between VAP and TAP, respectively.

Moreover, halophilic proteins generally require a high salt concentration to fold and enable enzymatic activity. We recently showed that AP from the moderate halophile *Halomonas* sp. 593 (HaAP) maintains enzymatic activity over a wide range of salt concentrations (1–4 *M* NaCl; Ishibashi *et al.*, 2005 ▶, 2011 ▶). It is known that halophile proteins are soluble in high-salt conditions owing to the affinity of surface carboxylates (provided by Glu and Asp residues) for solvated cations (a moderate salt concentration also effectively shields electrostatic repulsion of such surface negative charges); high salt also stabilizes the hydrophobic core by the salting-out effect. Indeed, HaAP shows a high acidic residue content [(Asp + Glu)/(Arg + Lys) = 83/30]. The sequence identity of HaAP is 70% compared with VAP, 34% compared with HsAP and 33% compared with TAP. The acidic residue content of HaAP (a moderate halophile) is higher than that of HsAP (an extreme halophile), whereas the amino-acid sequence of HaAP is most similar to that of VAP (a slight halophile). Thus, the adaptation mechanism of HaAP to a wide salt-concentration range cannot be explained only by the high acidic residue content (which is a high-salt adaptation).

In this study, to clarify the molecular mechanism for the adaptation of HaAP to function over a wide range of halophile environments, the tertiary structure of HaAP was determined to 2.1 Å resolution by X-ray crystallography. A structural comparison with other halophilic APs (HsAP, VAP and TAP) and non-halophilic AP from *Escherichia coli* (EcAP; PDB entry 1ed9; Stec *et al.*, 2000 ▶) revealed that unique structural characteristics exist in HaAP (*i.e.* the distribution of acidic residues and hydrophobic residues), with contrasting implications for solubility and stability in a salt environment, and are postulated to be responsible for the wide-range salt-concentration adaptation of HaAP.

## 2. Materials and methods   

### 2.1. Expression of HaAP   

HaAP was expressed in *E. coli* BL21 StarTM (DE3) pLysS cells as reported previously (Ishibashi *et al.*, 2011 ▶). In brief, the DNA fragment encoding the mature region of HaAP was amplified by PCR and then ligated into *Nde*I/*Bam*HI-digested pET3a to construct pHA.

*E. coli* BL21 Star (DE3) pLysS cells harbouring the pHA plasmid were grown in LB–ampicillin–chloramphenicol containing 0.4% glucose at 37°C overnight, and the 1% culture was added to 100 ml LB–ampicillin. After the OD_600_ had reached 0.8 at a cell-culture temperature of 18°C, synthesis of HaAP was induced for 8 h by the addition of 0.2 m*M* isopropyl-β-d-1-thiogalactopyranoside. Cells were disrupted in 20 ml ice-cold 50 m*M* Tris–HCl pH 8.0, 2 m*M* MgCl_2_ buffer (without NaCl) by sonication (SMT UH-150 sonifier with a 5 mm tip) for 3 min with a 40% pulse, and soluble and pellet fractions were obtained by centrifugation at 12 000*g* for 15 min.

### 2.2. Purification of recombinant HaAP   

The first step of protein purification by anion-exchange chromatography was performed as described previously (Ishibashi *et al.*, 2005 ▶, 2011 ▶). The soluble fraction of disrupted cells was applied onto a HiTrap Q HP column (1.6 × 2.5 cm, GE Healthcare) using an ÄKTAprime chromatography system (GE Healthcare). The bound proteins were eluted with a 100 ml linear gradient of NaCl from 0.3 to 0.9 *M* in 50 m*M* Tris–HCl pH 8.0, 2 m*M* MgCl_2_ buffer. The fractions containing HaAP were pooled and dialyzed against 50 m*M* Tris–HCl pH 8.0, 2 m*M* MgCl_2_ containing 3 *M* NaCl buffer. After dialysis, 30% ammonium sulfate was added and the soluble fraction was collected by centrifugation at 12 000*g* for 15 min. Proteins in the soluble fraction were loaded onto a hydrophobic column (30 ml, Toyopearl Phenyl-650M, Tosoh Bioscience) equilibrated with 50 m*M* Tris–HCl pH 8.0 containing 2 m*M* MgCl_2_, 0.5 *M* NaCl, 30% ammonium sulfate. The flowthrough fraction was then applied onto a gel-filtration column (1.6 × 60 cm, HiLoad Superdex 200 pg, GE Healthcare) equilibrated with 50 m*M* Tris–HCl pH 8.0, 2 m*M* MgCl_2_ buffer containing 0.5 *M* NaCl. The elution was pumped at 0.5 ml min^−1^. The protein purity was checked by 10% SDS–PAGE.

### 2.3. Crystallization of HaAP   

Screening for HaAP crystallization conditions was performed by the sitting-drop vapour-diffusion method using a 96-well Intelli-Plate (Hampton Research) and a Hydra II Plus One system (Matrix Technology) at 293 K. Before crystallization, HaAP was dialyzed against 50 m*M* Tris–HCl pH 8.0 containing 1 *M* NaCl and 2 m*M* SrCl_2_. A sitting drop was prepared by mixing 0.3 µl each of the protein solution and the reservoir solution, and the resulting drop was equilibrated against 70 µl reservoir solution. The search for crystallization conditions was performed using the commercially available kits Crystal Screen and Crystal Screen 2 (Hampton Research) and Wizard I and II (EmeraldBio). Cubic-shaped crystals of diffraction quality were obtained from Crystal Screen condition No. 6 [0.2 *M* MgCl_2_, 0.1 *M* Tris–HCl pH 8.5, 30%(*w*/*v*) PEG 4000] containing 15.0 mg ml^−1^ protein.

### 2.4. Diffraction experiments and structure determination   

Data sets were collected from HaAP crystals on beamlines BL5A and NW12A at the Photon Factory (PF), Tsukuba, Japan and on beamlines BL38B1 and BL41XU at SPring-8, Hyogo, Japan. Crystals were mounted on a nylon loop and cooled to −173°C in a nitrogen-gas stream. The HaAP crystal diffracted to 2.1 Å resolution and belonged to space group *P*2_1_. All collected data were integrated and scaled using the *HKL*-2000 suite of programs (Otwinowski & Minor, 1997 ▶). Diffraction data and processing statistics are shown in Table 1 ▶.

Initial phase information for HaAP was obtained by the molecular-replacement (MR) method using *MOLREP* (Vagin & Teplyakov, 2010 ▶), with the structure of VAP (PDB entry 3e2d; Helland *et al.*, 2009 ▶) as a search model. The modelling and refinement were carried out using *CNS* v.1.21 (Brünger *et al.*, 1998 ▶), *REFMAC*5 (Murshudov *et al.*, 2011 ▶) and *Coot* (Emsley *et al.*, 2010 ▶). The initial selection and manual adjustment of water-molecule positions were also performed with *Coot*. The data-refinement statistics are given in Table 1 ▶. According to the program *RAMPAGE* (Lovell *et al.*, 2003 ▶), 97.8% of the residues in the final model of HaAP are located in the favoured region of the Ramachandran plot and no residues are in the outlier region. The structural comparison and r.m.s. deviations of atoms were calculated using *LSQKAB* in the *CCP*4 package (Winn *et al.*, 2011 ▶) and the web-based program *PDBeFold* (http://www.ebi.ac.uk/msd-srv/ssm/; Krissinel & Henrick, 2004 ▶). Structural properties (hydrogen bonds, salt bridges, hydrophobic interactions, ASA, interface area *etc.*) at the molecular surface and at the interface of each AP were analyzed using the web-based program *PISA* (Krissinel & Henrick, 2007 ▶). Both the partial and the entire volumes of each AP were calculated using the web-based program 3*V* (Voss & Gerstein, 2010 ▶). The cavity volumes of each AP were calculated using *AVP* (Cuff & Martin, 2004 ▶). Metal ions in the crystal structure were identified based on the coordination geometry and analysis using the web-based program *STAN* (Nayal & Cera, 1996 ▶). Six Mg^2+^ ions and four Zn^2+^ ions were identified in an asymmetric unit (see §[Sec sec3]3). Ordered Na^+^ ions could not be identified in this study.

## 3. Results   

### 3.1. Overall structure of HaAP   

**Figure 1:**
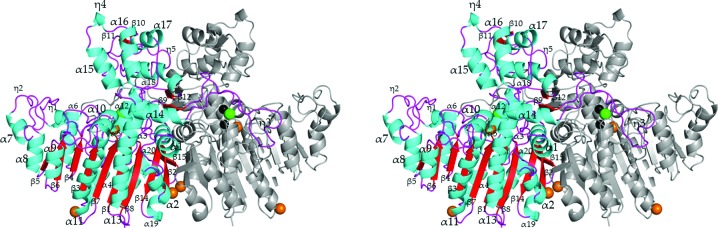
Stereoview of the dimeric unit of HaAP (a moderate halophile). The crown domain is located at the top of the figure. One monomer of the dimeric unit is coloured as follows; cyan, helix; red, β-strand; purple, loop. Zn^2+^, Mg^2+^ and Cl^−^ ions are shown by spheres coloured black, orange and green, respectively.

The crystal structure of HaAP was determined to 2.1 Å resolution with an *R* factor of 17.7% (*R*_free_ = 22.5%) in space group *P*2_1_, with unit-cell parameters *a* = 52.7, *b* = 147.0, *c* = 58.3 Å, β = 105.2° (Fig. 1 ▶). One asymmetric unit includes two HaAP chains (*A* and *B*) comprising 497 residues per chain, 93 water molecules, six Mg^2+^ ions, four Zn^2+^ ions and two Cl^−^ ions. Chains *A* and *B* in the asymmetric unit of the HaAP crystal are related by a noncrystallographic twofold axis. Electron density corresponding to the N-­terminal alanine in each chain was not visible.

The monomeric unit of HaAP consists of a domain with a β-sheet core (the ‘core’ domain, comprising residues Glu2–Thr314 and Thr464–Glu498) and a ‘crown’ domain (comprising residues Gly315–His463). The core domain involves 11 β-strands (β1, β2, β3, β4, β5, β6, β7, β8, β9, β14 and β15) with 19 surrounding helices (α1, α2, α3, α4, α5, α6, η1, η2, α7, α8, α9, α10, α11, α12, α13, α14, α19, η6 and α20). The crown domain consists of seven helices (η3, α15, η4, α16, α17, η5 and α18), four β-­strands (β10, β11, β12 and β13) and an extended ‘arm’ (Tyr321–Phe348) which wraps around the other monomer.

The crown domain is the most conspicuous structure in APs owing to its variable size (Du *et al.*, 2001 ▶; Hoylaerts *et al.*, 2006 ▶; Helland *et al.*, 2009 ▶; Supplementary Fig. S1^1^). The sizes of the crown domains of HaAP (149 residues, volume of 24 831 Å^3^) and VAP (149 residues, volume of 25 789 Å^3^) are similar and are significantly larger than those of HsAP (88 residues, volume of 13 872 Å^3^), TAP (35 residues, volume of 4778 Å^3^) and EcAP (42 residues, volume of 6736 Å^3^). The crown domains in HaAP and VAP also contain the extended arm.

Although the overall structure of the core domain is conserved among HaAP, VAP (a slight halophile), HsAP (an extreme halophile), TAP (a slight halophile) and EcAP (a non-halophile), the r.m.s.d. for C^α^ atoms of the core domain between HaAP and other APs is 0.88 Å for VAP, 1.34 Å for HsAP, 1.28 Å for TAP and 1.26 Å for EcAP. The main-chain structure of the monomer unit of HaAP is most similar to that of VAP (r.m.s.d. for C^α^ atoms of 0.82 Å, as shown in Table 2 ▶). Structural differences in APs are mostly attributed to the region of the crown domain, including several insertions in the primary structure of HaAP and VAP (Supplementary Fig. S1). The r.m.s.d.s for C^α^ atoms of the crown domain between HaAP and VAP are small (0.61 Å), whereas the r.m.s.d.s between HaAP and other APs are obviously larger: 2.84 Å for HsAP, 2.85 Å for TAP and 2.82 Å for EcAP. The structure of the extended arm inserted in HaAP (Tyr321–Phe348) and VAP (Tyr325–Phe352) (Supplementary Fig. 1 ▶) is also very similar (r.m.s.d. for main-chain atoms of 0.32 Å).

### 3.2. Structural characteristics of the molecular surface of HaAP   

**Figure 2:**
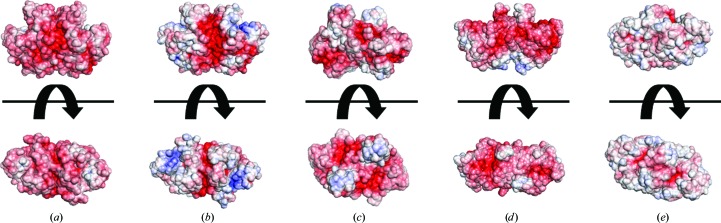
Electrostatic surface potentials of the dimeric units of (*a*) HaAP (a moderate halophile), (*b*) VAP (a slight halophile), (*c*) HsAP (an extreme halophile), (*d*) TAP (a slight halophile) and (*e*) EcAP (a non-halophile). The electrostatic surface potentials are contoured from −8*kT*/*q* (red) to 8*kT*/*q* (blue). This figure was created using the *APBS* plugin (Baker *et al.*, 2001 ▶) in *PyMOL* (http://www.pymol.org).

The molecular surface of HaAP consists of 620 solvent-accessible residues per dimer and the accessible surface area (ASA) was calculated to be 32 630 Å^2^ per dimer. When the surface amino-acid residues are classified into polar residues (Asp, Glu, Arg, Lys, His, Asn, Gln, Ser, Thr, Tyr and Cys) and nonpolar residues (Gly, Ala, Val, Leu, Ile, Pro, Phe, Met and Trp) (Timberlake, 1992 ▶), the numbers of solvent-accessible polar and nonpolar residues (ASA > 0 Å^2^) are 366 and 254, respectively. The ASAs of these polar and nonpolar residues are 21 245 and 11 385 Å^2^, corresponding to 59.0 and 41.0% of the total ASA, respectively. Polar residues at the surface of the HaAP dimer include 144 acidic residues (62 Asp and 82 Glu) and 52 basic residues (32 Arg and 20 Lys), corresponding to 23.2 and 8.4% of the total ASA, respectively. The number of negative charges [(Asp + Glu) − (Arg + Lys)] at the surface of dimeric HaAP was calculated to be 92. From the ASA and the number of negative charges described above, the negative charge density at the molecular surface of HaAP was calculated to be 2.8 × 10^−3^ e Å^−2^ (Table 2 ▶). Owing to the high density of negative charges at the surface of HaAP, the surface of HaAP is largely occupied by negative charges, even in comparison with other halophilic APs (Fig. 2 ▶).

The number of negative charges at the surface of HaAP (a moderate halophile) was compared with those of VAP (a slight halophile), HsAP (an extreme halophile), TAP (a slight halophile) and EcAP (a non-halophile). The numbers of negative charges at the dimer surfaces were calculated to be 22 for VAP, 60 for HsAP, 16 for TAP and ten for EcAP, which were lower than the 92 for HaAP. From the ASAs and the numbers of negative charges described above, the negative charge densities at the molecular surface were calculated to be 0.6 × 10^−3^ e Å^−2^ for VAP (a slight halophile), 1.9 × 10^−3^ e Å^−2^ for HsAP (an extreme halophile), 0.7 × 10^−3^ e Å^−2^ for TAP (a slight halophile) and 0.4 × 10^−3^ e Å^−2^ for EcAP (a non-halophile) (Table 2 ▶). Thus, the negative charge density at the surface of HaAP (2.8 × 10^−3^ e Å^−2^) is significantly higher than those of VAP, HsAP, TAP and EcAP.

### 3.3. Structural characteristics of the monomer–monomer interface   

As shown in Table 2 ▶, the interface between chain *A* and chain *B* of HaAP involves 120 nonpolar residues and 106 polar residues. The interface area is calculated to be 4155 Å^2^ and corresponds to 12.7% of the total ASA of the HaAP dimer. The interface contacts within 3.9 Å consist of 35 residues in six helix regions (α1, α2, α12, α18, η3 and η5), 17 residues in six β-­strand regions (β2, β9, β12, β13, β14 and β15) and 40 residues in 11 loop regions (Thr55–Asp64, Gly75–Ser80, Ala121–Leu131, Tyr321–Gly334, Ala338–Phe351, Val414–Glu423, Ala427–Phe431, Tyr435–Glu439, Gly457–Thr460, Gly472–Pro473 and Ser481–Ser482), as estimated using the web-based program *PISA* (Krissinel & Henrick, 2007 ▶). These contacts involve 60 hydrogen bonds and four salt bridges, as listed in Supplementary Table S1. Of these 60 hydrogen bonds, 18 were formed between main-chain atoms, 29 were formed between main-chain and side-chain atoms, and 13 were formed between side-chain atoms. These compositions of intermolecular hydrogen bonds were slightly different from those of VAP (a slight halophile); in the case of VAP, 20 were formed between main-chain atoms, 24 were formed between main-chain and side-chain atoms, and 15 were formed between side-chain atoms. 41 out of the total of 60 hydrogen bonds are composed of residues in the crown domain and 14 out of 41 hydrogen bonds are created by residues located in the extended arm (Supplementary Table S1). The intermolecular hydrogen bonds, consisting of four between main-chain atoms, six between main-chain atoms and side-chain atoms, and four between side-chain atoms (Supplementary Table S1), are almost conserved in VAP (four between main-chain atoms, seven between main-chain atoms and side-chain atoms, and two between side-chain atoms); this is only found in HaAP and VAP and is not observed in other APs (HsAP, TAP and EcAP).

### 3.4. Structure of catalytic site residues in HaAP   

**Figure 3:**
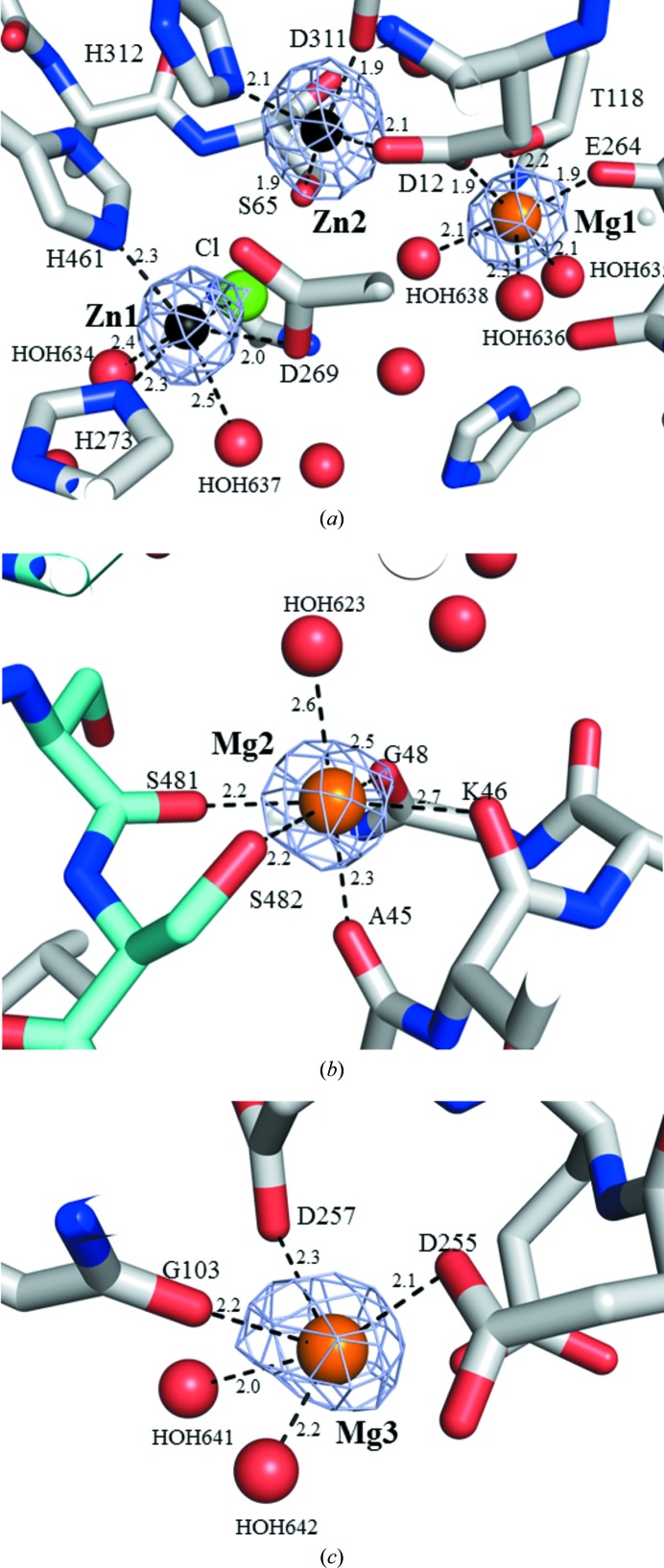
Metal ion-binding sites identified for HaAP. (*a*) Zn^2+^-binding sites (M1 and M2) and Mg^2+^-binding sites (M3) around the active site. (*b*) Mg^2+^-binding sites (M4) at the monomer–monomer interface. Chain *A* and chain *B* are coloured cyan and grey, respectively. (*c*) Mg^2+^-binding sites (M5) at the surface of a single chain. The mesh shows the *F*_o_ − *F*_c_ OMIT map within a +3σ contour level. Zn^2+^, Mg^2+^, Cl^−^ and the O atom of water are shown by spheres coloured black, orange, green and red, respectively.

The role of catalytic site residues in AP has been elucidated in detail using *E. coli* AP (EcAP; Kim & Wyckoff, 1991 ▶; Stec *et al.*, 2000 ▶). The catalytic site of EcAP is composed of two residues (Ser102 and Arg166), two Zn^2+^-binding sites (M1 and M2 sites) and one Mg^2+^-binding site (M3 site). The side chain of Arg166 binds the substrate, and the activated hydroxyl group of Ser102 attacks the phosphorus centre of the substrate (Stec *et al.*, 2000 ▶). The catalytic residues Ser102 and Arg166 in EcAP are conserved as Ser65 and Arg129, respectively, in HaAP. The r.m.s.d. for all atoms of the catalytic site residues between HaAP and other APs was 0.56 Å for VAP, 0.63 Å for HsAP, 0.54 Å for TAP and 0.31 Å for EcAP. The M1 site residues Asp327, His331 and His412 in EcAP are conserved as Asp269, His273 and His461 in HaAP (Fig. 3 ▶ *a*), in which Zn^2+^ (Zn1) chelates to three O atoms (O^δ2^ of Asp269 and two water O atoms) and two N atoms (N^∊2^ of His273 and N^∊2^ of His461) with distances of 2.1–2.5 Å (Supplementary Table S2). The M2 site residues Asp51, Ser102, Asp369 and His370 in EcAP are conserved as Asp12, Ser65, Asp311 and His312 in HaAP, in which Zn^2+^ (Zn2) chelates five O atoms (O^δ2^ of Asp12, O^γ^ of Ser65, O^δ2^ of Asp311 and two water O atoms) and one N atom (N^∊2^ of His312) with distances of 2.0–2.1 Å. The M3 site residues Asp51, Thr155 and Glu322 in EcAP are conserved as Asp12, Thr118 and Glu264 in HaAP, in which Mg^2+^ (Mg1) chelates six O atoms (O^δ1^ of Asp12, O^γ1^ of Thr118, O^∊2^ of Glu264 and three water O atoms) with distances of 1.9–2.3 Å.

**Figure 4:**
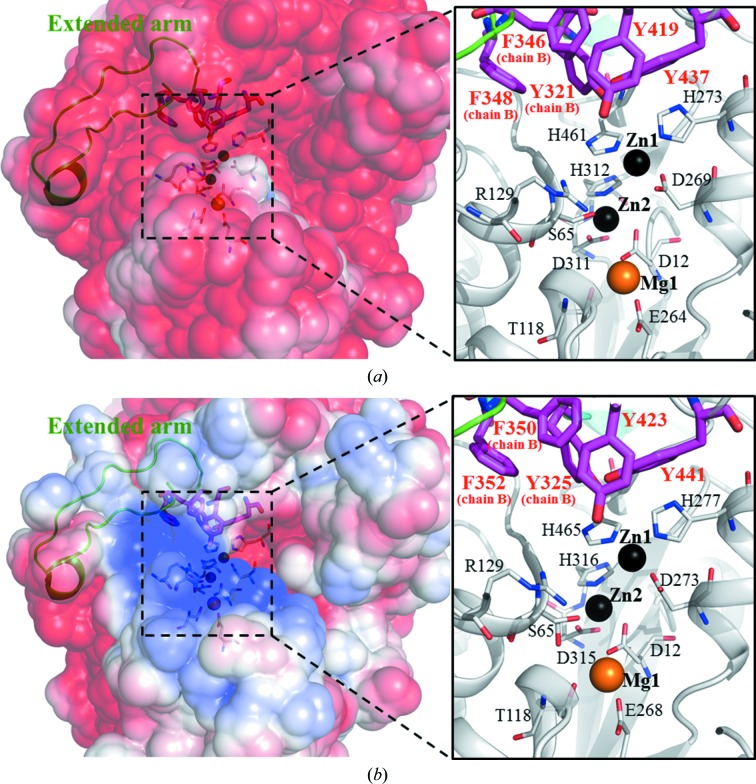
Electrostatic surface potentials and hydrophobic clusters near the catalytic sites of (*a*) HaAP and (*b*) VAP. In both (*a*) and (*b*) the electrostatic surface potentials are contoured from −3*kT*/*q* (red) to 3*kT*/*q* (blue). Green loops indicate the extended arms. In the enlargement on the right, residues in a hydrophobic cluster (with red labels) and in the catalytic site composed of Ser102 and Arg129 with residues in the M1, M2 and M3 sites (with black labels) are shown by bold magenta sticks and thin grey sticks, respectively.

In the vicinity of the M1 site of HaAP, we also found a hydrophobic cluster composed of five aromatic amino acids: Tyr419 and Tyr437 (in the crown domain of chain *A*), Tyr321, Phe346 and Phe348 (in the extended arm of chain *B*) (Fig. 4 ▶ *a*). The aromatic ring of Tyr321 in the hydrophobic cluster forms van der Waals contacts (within 4 Å distance) with the imidazole ring of His461 involved in the M1 site of the catalytic region of HaAP, as observed in halophilic APs (Tyr321 in HaAP, Tyr325 in VAP, Tyr360 in HsAP and Tyr325 in TAP) but not observed in EcAP.

### 3.5. Metal ion-binding sites that are newly observed in HaAP   

Two metal-binding sites (M4 and M5) were newly identified in the crystal structure of HaAP (Supplementary Table S2 and Figs. 3 ▶ *b* and 3 ▶ *c*). The M4 site is located at the interface between chain *A* and chain *B* in dimeric HaAP (Fig. 3 ▶ *b*). The M4 site is composed of six O atoms (four O atoms from the main chains of Ala45, Lys46, Gly48 and Ser481, the O^γ^ atom of Ser482 and one water O atom) chelating to a metal ion with distances of 2.2–2.7 Å (average distance of 2.4 Å; Supplementary Table S2).

The M5 site is located on the surface of each monomer of HaAP (Fig. 3 ▶ *c*). The M5 site is composed of at least five chelating O atoms (three O atoms from the main chain of Gly103, O^δ1^ of Asp255 and O^δ2^ of Asp257 and two O atoms from water molecules; Supplementary Table S2). A candidate for the sixth chelating O atom may be O^∊2^ of Glu256 located at a relatively large distance (3.2 Å). The average distances (excluding O^∊2^ of Glu256) to the chelating O atoms in the M5 site were 2.4 Å in chain *A* and 2.2 Å in chain *B*, which are similar to the typical Mg—O distance (1.9–2.3 Å) and shorter than the typical Sr—O distance (2.5–2.9 Å) (Shannon, 1976 ▶). Therefore, the observed metal ions in the M5 site were assigned as weakly bound Mg^2+^ and not as Sr^2+^.

## 4. Discussion   

### 4.1. Structural characteristics of HaAP   

It is known that APs hydrolyse aromatic and aliphatic phosphoesters and release phosphate; the hydrolysis reaction starts with the phosphate moiety binding to metal ions, Zn1 at the M1 site and Zn2 at the M2 site, and an arginine [Arg129 in HaAP and VAP (a slight halophile), Arg183 in HsAP (an extreme halophile), Arg148 in TAP (a slight halophile) and Arg166 in EcAP (a non-halophile)] (Stec *et al.*, 2000 ▶; de Backer *et al.*, 2002 ▶). The structures of the M1 site, the M2 site and the arginine are almost conserved between HaAP and other APs [VAP, HsAP, TAP and EcAP; the r.m.s.d.s of these M1 and M2 site residues and the arginine (a total of eight residues, see Supplementary Fig. S1) between HaAP and other APs are 0.22 Å for VAP, 0.32 Å for HsAP, 0.24 Å for TAP and 0.53 Å for EcAP]. However, the structure of the substrate entrance near the M1 site exhibits a large difference in these APs. For example, the M1 sites of HsAP, TAP and EcAP are largely exposed to solvent (the ASAs of the M1 site residues are 125.1 Å^2^ for HsAP, 123.0 Å^2^ for TAP and 84.6 Å^2^ for EcAP), whereas the M1 sites of the other APs (HaAP and VAP) are significantly less exposed (the ASAs of the M1 sites are 66.2 and 68.3 Å^2^, respectively), principally owing to residues (Tyr321 and Tyr437 in HaAP and Tyr325 and Tyr441 in VAP) in the hydrophobic cluster that partly cover the phosphate-binding site near the M1 site. A similar hydrophobic cluster composed of Tyr423 and Tyr441 (in the crown domain in chain *A*) and Tyr325, Phe350 and Phe352 (in the extended arm in chain *B*) are observed in VAP (Figs. 4 ▶ *a* and 4 ▶ *b*). Since this hydrophobic cluster does not exist in APs without the extended arm (HsAP, TAP and EcAP), the function of the extended arm may not only contribute to the association of HaAP and VAP monomers but also to the substrate specificity of HaAP and VAP compared with other APs (HsAP, TAP and EcAP, which lack the extended arm).

Another characteristic feature observed in the active site of HaAP is the lack of positive potential at the entrance to the active site of VAP, as shown in Figs. 4 ▶(*a*) and 4 ▶(*b*). A relatively large positive potential composed of Lys418 and Arg340 in the crown domain and Arg113, Arg129, Arg153, Lys177, Lys179, Arg180 and Lys181 in the β-sheet core domain induces the binding of negatively charged phospho esters in the active site of VAP (Helland *et al.*, 2009 ▶). The lack of positive potential at the entrance to the active site may reflect the adaptation of HaAP to a high concentration of salt. The hydrophobic cluster, rather than positive potential at the entrance, may be more important for HaAP to retain enzymatic activity at higher salt concentrations, which is supported by the experimental data that the enzymatic activity of HaAP increases according to the increase in the NaCl concentration (Ishibashi *et al.*, 2011 ▶).

### 4.2. Structural changes to increase the acidic surface in HaAP   

**Figure 5:**
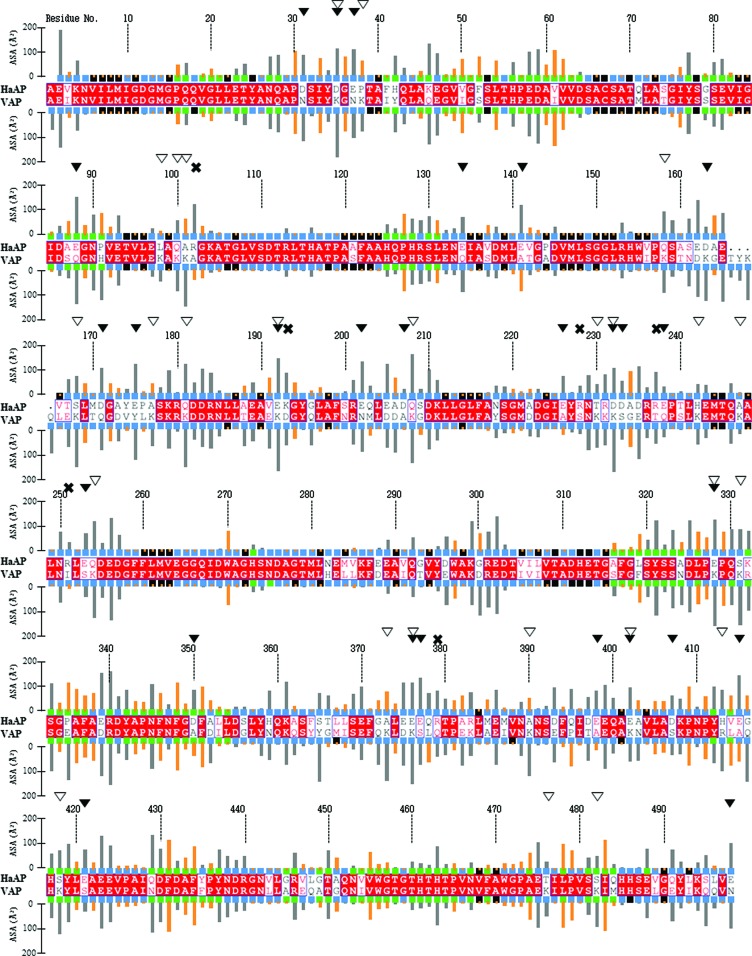
Amino-acid sequence alignment of HaAP (a moderate halophile) and VAP (a slight halophile, PDB entry 3e2d). Sequence homology is highlighted by red letters; sequence identity is shown as white letters on a red background. The boxes above and below the sequences indicate the locations of the residues in the AP monomers (blue, solvent-accessible residues; black, inaccessible residues; green, monomer–monomer interface residues). Grey and orange bars show the ASAs of polar and nonpolar residues, respectively. Filled triangles above the residue numbers show substitutions by Asp or Glu in HaAP. Open triangles above the residue numbers show substitution of Arg or Lys by other residues in HaAP. Crosses above the residue numbers show substitutions by Arg or Lys in HaAP.

A negative surface charge is a hallmark of halophilic proteins, promoting the binding of hydrated salt cations and thereby maintaining solubility in a high-­salt environment (Lanyi, 1974 ▶). Although the acidic residue content of HaAP (a moderate halophile) is higher than that of VAP (a slight halophile), the tertiary structure of HaAP is most similar to that of VAP. The differences in amino-acid composition between HaAP and VAP mainly appear at the molecular surface, since 166 surface residues, 23 interfacing residues and eight buried residues differ between HaAP and VAP (Fig. 5 ▶). From the structural comparison between HaAP and VAP, HaAP acquired more negative charges by 19 substitutions of non-acidic residues by Asp or Glu out of 116 surface-residue substitutions (*i.e.* Asp31, Asp171, Asp207, Asp233, Asp397, Asp407, Glu37, Glu134, Glu141, Glu175, Glu202, Glu226, Glu238, Glu253, Glu377, Glu398, Glu415, Glu421 and Glu498) and lost positive charges by 23 out of 36 substitutions of Lys by non-basic residues (*i.e.* Asp35, Pro38, Leu98, Gln100, Ala101, Gln158, Asp163, Ser168, Ala177, Gln181, Gln192, Gln208, Thr230, Asp232, Ala247, Gln254, Glu328, Ser331, Ala373, Glu376, Ala390, Gln402 and Thr476) (Fig. 5 ▶). Conversely, HaAP acquired positive charges by six substitutions of nonbasic residues by Arg or Lys (Arg102, Lys193, Arg228, Arg237, Arg251 and Arg379; Fig. 5 ▶). It was also found that the side chains of Arg102, Lys193, Arg237, Arg251 and Arg379 (but not Arg228) create new contacts (salt bridges) with the negatively charged side chains of Glu141, Asp207, Glu238, Glu376 and an O atom of the C-terminus of Glu498 within distances of less than 4.0 Å (Supplementary Table S3). In addition, the numbers of basic residues (Arg and Lys) forming salt bridges at the monomer surface were 17 for HaAP, 15 for VAP, ten for HsAP, 11 for TAP and 15 for EcAP (Supplementary Table S3), which are equivalent to 65, 33, 45, 44 and 47% of the surface basic residues, respectively. These results suggest that the positive charges of basic residues in HaAP are suppressed by the formation of salt bridges more effectively than in other APs. The simultaneous introduction of both positive and negative charged residues to form salt bridges might be beneficial to stabilize HaAP under low salt concentrations; also, the abundant negatively charged residues are beneficial for its solbilization at high salt concentrations.

### 4.3. The structural feature of buried amino acids in HaAP   

While a high content of acidic residues at the surface of halophilic proteins provides high solubility under high salt concentrations, it may also destabilize the structure of halophilic proteins under low salt concentrations owing to charge repulsion (although this would be effectively screened in >0.2 *M* salt). It is known that the relative activity of an alkaline phosphatase from the extreme halophile *Haloarcula marismortui* [HmAP; UniProt ID Q5V573, (Asp + Glu)/(Arg + Lys) = 2.74] gradually decreases with Na^+^ and Ca^2+^ concentration (Goldman *et al.*, 1990 ▶). If the relative activity of HmAP in the presence of 4 *M* NaCl and 3.5 m*M* CaCl_2_ is set to 100%, its relative activity in the presence of 1 *M* NaCl and 3.5 m*M* CaCl_2_ decreases to less than 30% (Goldman *et al.*, 1990 ▶). On the other hand, the enzymatic activity of HaAP is retained over a wide range of salt concentration (1–4 *M* NaCl). If the relative activity of HaAP in 3 *M* NaCl is set to 100%, about 60% of its relative activity remains in 1 *M* NaCl for at least 4 d (Ishibashi *et al.*, 2005 ▶, 2011 ▶).

In order to consider the reason why HaAP can adapt to a wide range of salt concentration, we focused on the differences in the structural characteristics of the inaccessible hydrophobic residues and the monomer–monomer interface between HaAP and other APs (VAP, HsAP, TAP and EcAP). The HaAP monomer involves 37 inaccessible hydrophobic residues (Val, Leu, Ile, Pro, Phe, Met and Trp showing an ASA of 0 Å^2^); this number is larger than those for VAP (24 residues), HsAP (24 residues), TAP (26 residues) and EcAP (27 residues), as shown in Table 2 ▶. The volumes of inaccessible hydrophobic residues in the monomers are 7850 Å^3^ for HaAP, 5707 Å^3^ for VAP, 5592 Å^3^ for HsAP, 6060 Å^3^ for TAP and 5876 Å^3^ for EcAP. Since the volumes of the monomers of HaAP, VAP, HsAP, TAP and EcAP were calculated to be 76 573, 80 555, 64 222, 51 173 and 67 942 Å^3^, respectively, the volume ratios of inaccessible hydrophobic residues are 10.3, 7.1, 8.7, 11.8 and 8.6%, respectively, suggesting that the content of inaccessible hydrophobic residues in HaAP is larger than those of other APs, with the exception of TAP (a slight halophile).

The hydrophobicity of the interior of HaAP was also evaluated by counting the numbers of C atoms. The numbers of C atoms of inaccessible residues are 329 for HaAP (a moderate halophile), 264 for VAP (a slight halophile), 250 for HsAP (an extreme halophile), 246 for TAP (a slight halophile) and 248 for EcAP (a non-halophile), as shown in Table 2 ▶. This result also suggests that the hydrophobicity of the interior of HaAP is predominantly greater than those of other APs.

Moreover, we also compared the cavity volumes in the interior of different APs. The cavity volumes calculated in the dimer units are 22 570 Å^3^ for HaAP, 31 544 Å^3^ for VAP, 22 317 Å^3^ for HsAP, 19 399 Å^3^ for TAP and 26 994 Å^3^ for EcAP, as shown in Table 2 ▶. The ratios of the cavity volume to the entire volume of the dimer unit are calculated to be 14.7% for HaAP, 19.6% for VAP, 17.4% for HsAP, 19.0% for TAP and 19.9% for EcAP, indicating that the atomic density in the whole of the HaAP dimer is greater than those of VAP, HsAP, TAP and EcAP (Table 2 ▶). From the ratios for the volumes of inaccessible hydrophobic residues and the ratios of the cavity volumes, the internal core of HaAP may be more stable than those of VAP, HsAP, TAP and EcAP, which may be part of the reason why HaAP is stable and functional under low-salt conditions.

The strength of the association of the monomer–monomer interface would contribute to the stability of the biological form of the protein and may also be part of the reason for the adaptation of HaAP to a wide salt-concentration range. Structural determination of HaAP showed that 60 hydrogen bonds and four salt bridges contribute to the dimerization of HaAP; the number of attractive hydrogen-bond interactions is similar to those in VAP (61 hydrogen bonds and 11 salt bridges) and EcAP (58 hydrogen bonds and ten salt bridges) and is predominantly larger than those in HsAP (32 hydrogen bonds and two salt bridges) and TAP (26 hydrogen bonds and no salt bridges) (Table 2 ▶). Moreover, the number of inter­facing hydrophobic residues of HaAP is 80 per dimer, which is similar to that in VAP (78 residues) and significantly larger than those in HsAP (30 residues), TAP (30 residues) and EcAP (50 residues). These numbers of interface hydrophobic residues are equivalent to 35.4, 33.9, 22.7, 29.4 and 24.5% of all interfacing residues, respectively. These results suggest that the interface hydrophobic interactions of HaAP (a moderate halophile) and VAP (a slight halophile) may be stronger than those of HsAP (an extreme halophile), TAP (a slight halophile) and EcAP (a non-halophile). In fact, the Δ_*i*_ *G* values reflecting the intensity of hydrophobic interaction at the monomer–monomer interface are estimated to be −52.2 kcal mol^−1^ for HaAP and −56.1 kcal mol^−1^ for VAP, which are about twice as large as those for HsAP (−21.4 kcal mol^−1^), TAP (−26.5 kcal mol^−1^) and EcAP (−29.1 kcal mol^−1^) (Table 2 ▶).

Overall, from analysis of the structural features, we propose that the adaptation of HaAP to a wide range of salt concentrations is likely to be achieved through a combination of negative surface charge density (a feature of extreme halophile proteins that enables solubility under high-salt conditions) as well as an abundant content of hydrophobic residues in the interior of HaAP and at the monomer–monomer interface (a feature of slight halophile proteins that enhances structural stability under low-salt conditions).

## Supplementary Material

PDB reference: HaAP, 3wbh

Supplementary Information.. DOI: 10.1107/S1399004713033609/mh5105sup1.pdf

## Figures and Tables

**Table 1:** Data-collection and refinement statistics for HaAP (a moderate halophile) Values in parentheses are for the highest resolution shell.

Wavelength ()	1.0000
Space group	*P*2_1_
Unit-cell parameters (, )	*a* = 52.7, *b* = 147.0, *c* = 58.3, = 90, = 105.2, = 90
Resolution ()	2.10 (2.142.10)
No. of unique reflections	48023
Multiplicity	2.6 (2.0)
*R*_merge_ † (%)	8.4 (33.5)
Completeness (%)	94.2 (87.2)
*I*/(*I*) (%)	9.3 (2.3)
*R* factor‡ (%)	17.7 (22.2)
*R*_free_ (%)	22.5 (27.1)
Mean *B* value (^2^)	19.2
No. of reflections used	45557
No. of protein atoms	7632
No. of waters	93
No. of inorganic ions	6 Mg^2+^, 4 Zi^2+^, 2 Cl
R.m.s.d. stereochemistry§	
Bond lengths ()	0.014
Bond angles ()	1.576
Ramachandran analysis¶ (%)	
Favoured regions	97.8
Allowed	2.2
Disallowed	0
PDB code	3wbh

† *R*_merge_ = 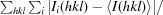 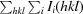.

‡ *R* factor and *R*_free_ = 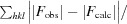 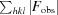, where the free reflections (5% of the total used) were held aside to calculate *R*_free_ throughout the refinement.

§ R.m.s.d. stereochemistry is the deviation from ideal values.

¶ Ramachandran analysis was carried out using *RAMPAGE* (Lovell *et al.*, 2003 ▶).

**Table 2:** Comparison of the structural characteristics of APs

	HaAP	VAP	HsAP	TAP	EcAP
Halophilicity	Moderate	Slight	Extreme	Slight	None
PDB code	3wbh	3e2d	2x98	2iuc	1ed9
Sequence identity (%)	100.0	70.0	33.6	32.9	32.7
R.m.s.d. for C atoms ()	0	0.82	1.90	1.29	1.41
Volume of dimer† (^3^)	153146	161110	128444	102346	135884
Cavity volume in dimer‡ (^3^)	22570	31544	22317	19399	26994
Volume ratio of cavity to dimer (%)	14.7	19.6	17.4	19.0	19.9
ASAs					
ASA of dimer (^2^)	32630	34470	32304	22410	27920
ASA of nonpolar residues (^2^)	11385	10325	14115	7143	10440
ASA of polar residues (^2^)	21245	24145	18189	15267	17480
Amino-acid composition of dimer					
Nonpolar/polar residues§	1.02 (504/492)	0.87 (466/538)	1.05 (440/420)	0.95 (366/384)	1.02 (454/446)
Acidic residues (Asp + Glu)	72 + 94	68 + 66	72 + 54	44 + 40	56 + 48
Basic residues (Arg + Lys)	36 + 24	26 + 76	42 + 12	12 + 58	28 + 56
(Asp + Glu)/(Arg + Lys)	2.77	1.31	2.33	1.20	1.24
Solvent-accessible surface residues (ASA > 0^2^) in dimer					
Nonpolar/polar residues§	0.69 (254/366)	0.66 (256/386)	0.88 (284/324)	0.70 (194/276)	0.86 (268/310)
Acidic residues (Asp + Glu)	62 + 82	58+ 56	54 + 50	36 + 30	32 + 42
Basic residues (Arg + Lys)	32 + 20	20 + 72	34 + 10	10 + 40	14 + 50
(Asp + Glu)/(Arg + Lys)	2.77	1.24	2.36	1.32	1.16
Density of negative charge (e^2^)	0.0028	0.0006	0.0019	0.0007	0.0004
Inaccessible residues (ASA = 0^2^) in monomer					
Total volume of inaccessible residues† (^3^)	13682	11540	10717	9869	10703
Total No. of inaccessible residues	74	66	58	54	58
Total No. of C atoms of inaccessible residues	329	264	250	246	248
Ratio of C atoms and volume	0.024	0.023	0.023	0.025	0.023
Nonpolar/polar residues§	6.40 (64/10)	2.88 (49/17)	8.67 (52/6)	5.75 (46/8)	4.80 (48/10)
No. of hydrophobic residues¶	37	24	24	26	27
Volume of inaccessible hydrophobic residues† (^3^)	7850	5707	5592	6060	5876
Volume ratio of inaccessible hydrophobic residues to monomer (%)	10.3	7.1	8.7	11.8	8.6
Monomermonomer interface					
Interface area (^2^)	4155	4270	2207	1825	3807
No. of interfacing residues	226	230	132	102	204
Nonpolar/polar residues†	1.13 (120/106)	0.95 (112/118)	0.61 (50/82)	0.76 (44/58)	0.79 (90/114)
No. of interfacing hydrophobic residues	80	78	30	30	50
No. of hydrogen bonds	60	61	32	26	58
No. of salt bridges	4	11	2	0	10
_*i*_ *G* †† (kcalmol^1^)	52.2	56.1	21.4	26.5	29.1

† The web-based program 3*V* was used for this calculation (Voss Gerstein, 2010 ▶).

‡ *AVP* was used for this calculation (Cuff Martin, 2004 ▶). This calculation not only includes cavity volumes in the two monomers but also in the monomermonomer interface.

§ Nonpolar residues are Gly, Ala, Val, Leu, Ile, Pro, Phe, Met and Trp. Polar residues are Asp, Glu, Arg, Lys, His, Asn, Gln, Ser, Thr, Tyr and Cys.

¶ Hydrophobic residues are Val, Leu, Ile, Pro, Phe, Met and Trp.

†† _*i*_ *G* indicates the hydrophobic interactions in the monomermonomer interface as calculated by *PISA* (Krissinel Henrick, 2007 ▶).

## Footnotes

1 Supporting information has been deposited in the IUCr electronic archive (Reference: MH5105).
