# Supplementary material for: Structural characteristics of alkaline phosphatase from the moderately halophilic bacterium Halomonas sp. 593
Source: Acta Crystallogr D Biol Crystallogr. 2014 Feb 22;70(Pt 3):811–20. doi: 10.1107/S1399004713033609 (PMC3949524; doi:10.1107/S1399004713033609)
Supplement: Supplementary file 1 [file d-70-00811-sup1.pdf]

Supporting Information

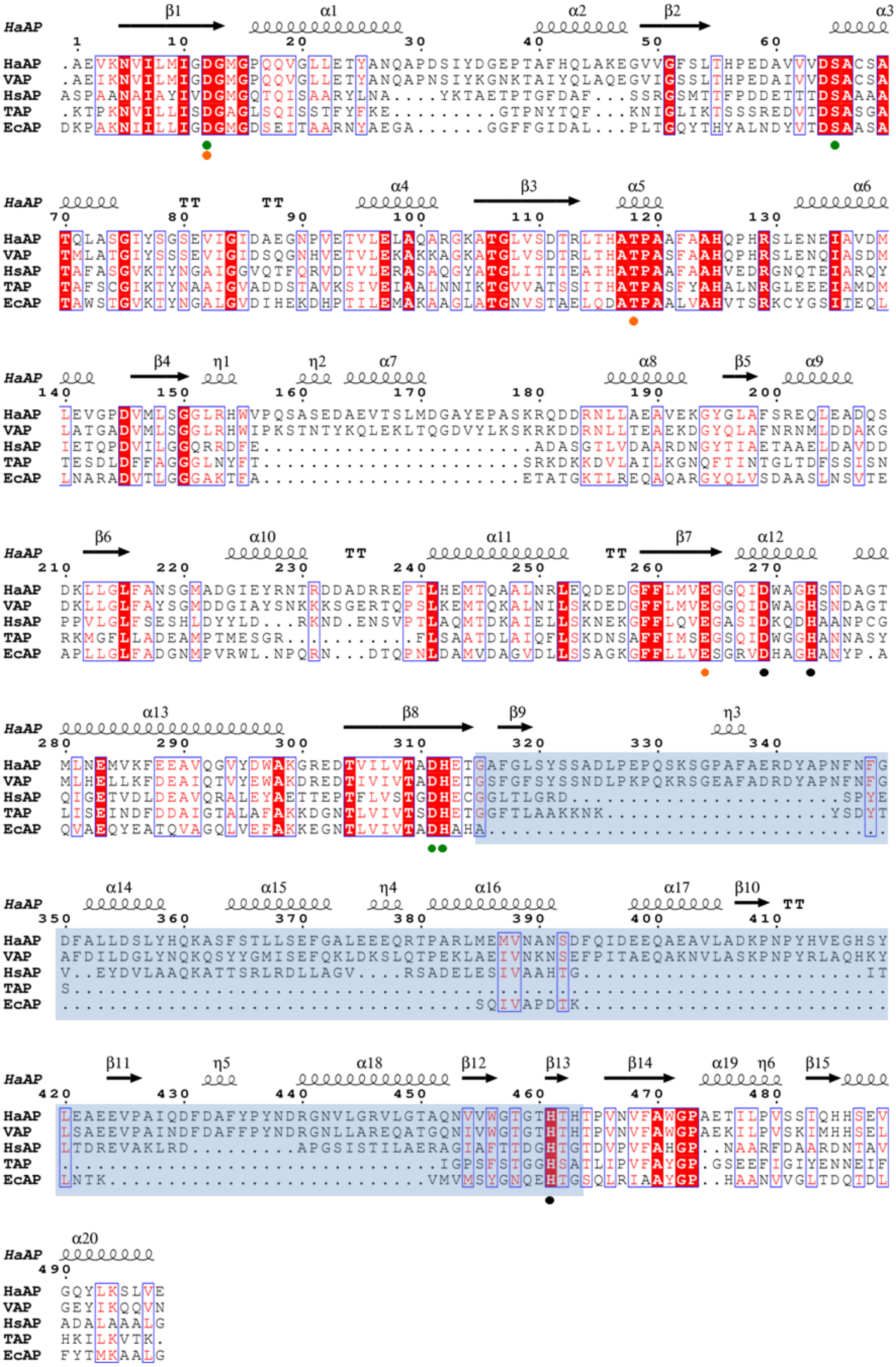

Supporting Figure S1. Structural alignment generated by pairwise comparison of the structure of HaAP (moderate halophile), VAP (slight halophile, 3E2D), HsAP (extreme halophile, 2X98), TAP (slight halophile, 2IUC) and EcAP (non-halophile, 1ED9) using the Dali server (http://ekhidna.biocenter.helsinki.fi/dali_server). Sequence homologies highlighted in red letters; sequence identities are shown as white letters on red background. The region shown in a blue background indicates the “crown” domain. Black, green and orange circles under letters indicate the residues involved in chelation in M1, M2, and M3 sites, respectively.

Supporting Table S1. Hydrogen bonds and salt bridges at the monomer-monomer interface of HaAP (moderate halophile).

Location	No.	Residue	Atom	Distance(Å)	Atom	Residue	No.	Location
				**Hydrogen bonds**				
α1	17	Gln	O	2.9	Nε2	His	463	loop
α2	42	His	Nε2	3.3	O	Ile	483	β15
α2	46	Lys	O	3.6	Oγ	Ser	482	loop
β2	50	Val	N	3.9	O	Ser	481	loop
			N	3.1	Oγ			
			O	2.7	Oγ			
loop	59	Asp	Oδ2	2.8	Oη	Tyr	342	loop
loop	61	Val	N	3.1	O	Phe	348	loop
			N	2.9	O	Gly	349	loop
loop	62	Val	N	2.9	O	Phe	348	loop
β-turn	80	Ser	N	3.1	Oη	Tyr	342	loop
β-turn	81	Glu	Oε1	2.9	N	Asn	345	loop
β-turn	88	Glu	O	2.8	N	Gly	334	loop
loop	89	Gly	O	3.0	N	Ala	336	η3
β9	319	Leu	O	3.9	O	His	461	loop
			O	3.7	Nδ1	His	463	loop
			O	3.0	N			
loop	320	Ser	Oγ	2.8	Oγ1	Thr	458	loop
			Oγ	2.6	Oγ1	Thr	460	loop
			Oγ	2.9	O	His	461	β13
loop	321	Tyr	Oη	2.5	Oη	Tyr	437	loop
			N	3.5	Oγ1	Thr	460	loop
			O	2.7	Oγ1			
			N	3.6	O	His	461	β13
loop	323	Ser	N	2.8	O	Ala	433	η5
			Oγ	3.1	O			
loop	333	Ser	N	3.8	O	Glu	88	β-turn
			Oγ	3.2	Oε1			
η3	336	Ala	N	2.7	O	Gly	89	β-turn
loop	342	Tyr	Oη	3.2	Oδ2	Asp	59	loop

			Oη	2.7	N	Ser	80	β-turn
			Oη	3.7	Oγ	Ser	80	β-turn
loop	345	Asn	N	2.8	Oε1	Glu	81	β-turn
			O	2.8	Nε2	His	417	loop
loop	348	Phe	O	3.3	N	Val	61	loop
			O	3.0	N	Val	62	loop
loop	349	Gly	O	3.1	N	Val	61	loop
loop	417	His	Nε2	2.8	O	Asn	345	loop
			Nε2	3.9	Oδ1			
loop	430	Asp	Oδ2	3.6	N	Gln	429	loop
			N	2.9	Oδ2	Asp	430	loop
			Oδ2	2.7	N			
			O	2.9	Nε	Arg	446	α18
η5	433	Ala	O	2.9	N	Ser	323	loop
			O	3.3	Oγ			
loop	437	Tyr	Oη	2.5	Oη	Tyr	321	loop
α18	446	Arg	Nε	3.2	Oδ2	Asp	430	loop
α18	458	Thr	Oγ1	2.6	Oγ	Ser	320	loop
loop	460	Thr	Oγ1	2.7	Oγ			
			Oγ1	2.8	O	Tyr	321	loop
			Oγ1	3.5	N			
β13	461	His	O	3.6	N			
loop	463	His	Nε2	3.0	O	Gln	17	α1
			N	2.9	O	Leu	319	β9
			Nδ1	3.6	O			
loop	481	Ser	Oγ	3.3	O	Val	50	β2
			Oγ	3.0	N			
			Oγ	2.8	Oγ	Ser	481	loop
loop	482	Ser	Oγ	3.3	O	Ala	45	α2
			Oγ	3.7	O	Lys	46	α2
				**Salt bridges**				
α18	446	Arg	Nε	2.8	Oδ2	Asp	430	loop
			Nη2	3.1	Oδ2			
loop	430	Asp	Oδ2	2.9	Nε	Arg	446	α18
			Oδ2	3.3	Nη2			

Supporting Table S2. Metal ions observed in HaAP crystal.

Site	Atom	Chain	No.	Distance (Å)	Atom	Residue	No.	Chain
M1	Zn1	A	504	2.3	Oδ2	Asp	269	A
				2.2	Nε2	His	273	A
				2.3	Nε2	His	461	A
				2.5	O	HOH	633	A
				2.6	O	HOH	636	B
M2	Zn2	A	505	2.1	Oδ2	Asp	12	A
				1.7	Oγ	Ser	65	A
				2.1	Oδ2	Asp	311	A
				2.0	Nε2	His	312	A
M3	Mg1	A	501	2.0	Oδ2	Asp	12	A
				2.1	Oγ1	Thr	118	A
				1.9	Oε2	Glu	264	A
				2.1	O	HOH	638	A
				2.2	O	HOH	639	A
				2.2	O	HOH	640	A
M4	Mg2	A	502	2.2	O	Ala	45	A
				2.4	O	Lys	46	A
				2.2	O	Gly	48	A
				2.4	O	Ser	481	B
				2.5	Oγ	Ser	482	B
				2.4	O	HOH	637	B
M5	Mg3	A	503	2.5	O	Gly	103	A
				2.3	Oδ1	Asp	255	A
				2.5	Oδ2	Asp	257	A
				2.7	O	HOH	634	A
				2.0	O	HOH	646	A
M1	Zn1	B	504	2.0	Oδ2	Asp	269	B
				2.3	Nε2	His	273	B
				2.3	Nε2	His	461	B
				2.4	O	HOH	634	A
				2.5	O	HOH	637	B

M2	Zn2	B	505	2.1	Oδ1	Asp	12	B
				1.9	Oγ	Ser	65	B
				1.9	Oδ2	Asp	311	B
				2.1	Nε2	His	312	B
M3	Mg1	B	501	1.9	Oδ2	Asp	12	B
				2.2	Oγ1	Thr	118	B
				1.9	Oε2	Glu	264	B
				2.1	O	HOH	635	B
				2.3	O	HOH	636	B
				2.1	O	HOH	638	B
M4	Mg2	B	502	2.2	O	Ser	481	A
				2.2	Oγ	Ser	482	A
				2.3	O	Ala	45	B
				2.7	O	Lys	46	B
				2.5	O	Gly	48	B
				2.6	O	HOH	623	A
M5	Mg3	B	503	2.2	O	Gly	103	B
				2.1	Oδ1	Asp	255	B
				2.3	Oδ2	Asp	257	B
				2.0	O	HOH	641	B
				2.2	O	HOH	642	B

Supporting Table 3. Comparison of salt bridges on the monomer surface of APs.

No.	Residue	Atom	Distance (Å)	Atom	Residue	No.
			**HaAP (this work)**			
47	Glu	Oε2	3.6	Nξ	Lys	299
111	Asp	Oδ2	3.0	Nξ	Lys	286
132	Glu	Oε2	2.7	Nη2	Arg	113
141	Glu	Oε1	4.0	Nξ	Lys	193
182	Asp	Oδ2	2.8	Nη2	Arg	180
189	Glu	Oε1	2.8	Nη2	Arg	184
207	Asp	Oδ2	2.9	Nη2	Arg	251
238	Glu	Oε2	3.8	Nη1	Arg	237
243	Glu	Oε2	3.6	Nη2	Arg	201
243	Glu	Oε1	2.8	Nη1	Arg	236
283	Glu	Oε1	2.7	Nξ	Lys	286
289	Glu	Oε2	2.9	Nη1	Arg	231
303	Asp	Oδ2	2.7	Nη2	Arg	301
376	Glu	Oε1	2.9	Nη2	Arg	379
407	Asp	Oδ1	2.6	Nη2	Arg	440
432	Asp	Oδ2	2.6	Nξ	Lys	408
498	Glu	O	3.6	Nη2	Arg	102
			**HsAP (2X98)**			
93	Asp	Oδ2	2.8	Nη1	Arg	67
114	Asp	Oδ2	2.8	Nη1	Arg	183
188	Glu	Oε2	2.9	Nη2	Arg	191
195	Glu	Oε1	3.0	Nη1	Arg	191
196	Glu	Oε2	3.0			
211	Glu	Oε2	2.4	Nη1	Arg	207
253	Glu	Oε2	3.0	Nη1	Arg	208
265	Asp	Oδ1	4.0	Nη2	Arg	376
317	Glu	Oε2	3.7	Nη2	Arg	262
320	Glu	Oε1	2.8			
407	Glu	Oε1	2.8	Nη2	Arg	425
424	Glu	Oε1	3.6	Nη2	Arg	356
457	Asp	Oδ1	2.7	Nξ	Lys	127
			**VAP (3E2D)**			

93	Glu	Oε1	2.9	Nξ	Lys	101
111	Asp	Oδ2	2.8	Nξ	Lys	290
132	Glu	Oε1	2.9	Nη2	Arg	113
186	Asp	Oδ2	2.9	Nη2	Arg	184
193	Glu	Oε1	2.8	Nη2	Arg	188
226	Asp	Oδ2	2.8	Nξ	Lys	234
247	Glu	Oε1	2.9	Nη1	Arg	240
247	Glu	Oε2	2.8	Nη2	Arg	240
287	Glu	Oε1	2.7	Nξ	Lys	290
293	Glu	Oε2	2.8	Nξ	Lys	235
300	Glu	Oε1	2.8	Nξ	Lys	303
307	Asp	Oδ2	2.9	Nη2	Arg	305
390	Glu	Oε2	2.7	Nξ	Lys	387
397	Glu	Oε2	3.9	Nξ	Lys	365
436	Asp	Oδ2	2.7	Nξ	Lys	412
**TAP (2IUC)**						
83	Asp	Oδ2	3.1	Nη1	Arg	148
106	Asp	Oδ1	3.4	Nη2	Arg	78
164	Asp	Oδ1	2.9	Nη1	Arg	209
179	Asp	Oδ2	3.1	Nη2	Arg	177
273	Glu	Oε1	2.9	Nη1	Arg	227
276	Asp	Oδ2	2.7	Nη2	Arg	227
278	Asp	Oδ2	2.5	Nξ	Lys	57
279	Asp	Oδ1	2.9	Nξ	Lys	57
353	Glu	Oε2	3.3	Nξ	Lys	366
353	Glu	Oε1	3.3	Nξ	Lys	369
359	Glu	Oε1	2.8	Nξ	Lys	96
**EcAP (1ED9)**						
529	GLN	Oε1	3.1	Nη1	Arg	510
539	Asp	Oδ1	3.4	Nξ	Lys	540
544	ASN	Oδ1	3.9	Nξ	Lys	543
576	Asp	Oδ1	2.8	Nη1	Arg	562
601	Asp	Oδ1	3.0	Nη1	Arg	666
626	Glu	Oε1	2.8	Nξ	Lys	591
628	Asp	Oδ1	2.5	Nξ	Lys	688
653	Asp	Oδ2	2.8	Nξ	Lys	828

697	ASN	Oδ1	2.7	Nη1	Arg	732
728	GLN	Oε1	3.1			
751	GLN	Oε1	2.9	Nξ	Lys	637
808	Glu	Oε1	3.2	Nξ	Lys	805
			3.8	Nξ	Lys	812
844	Asp	Oδ2	3.2	Nη1	Arg	767
847	Glu	Oε2	2.4	Nη2	Arg	767
854	Glu	Oε1	3.6	Nη2	Arg	851
859	Glu	Oε1	4.0	Nξ	Lys	543
934	Asp	Oδ1	2.7	Nξ	Lys	614
